# Minimizing the Total Service Time of Discrete Dynamic Berth Allocation Problem by an Iterated Greedy Heuristic

**DOI:** 10.1155/2014/218925

**Published:** 2014-09-08

**Authors:** Shih-Wei Lin, Kuo-Ching Ying, Shu-Yen Wan

**Affiliations:** ^1^Department of Information Management, Chang Gung University, 259 Wen-Hwa 1st Road, Kwei-Shan, Taoyuan 333, Taiwan; ^2^Department of Industrial Engineering and Management, National Taipei University of Technology, No. 1, Section 3, Chung-Hsiao East Road, Taipei 10608, Taiwan

## Abstract

Berth allocation is the forefront operation performed when ships arrive at a port and is a critical task in container port optimization. Minimizing the time ships spend at berths constitutes an important objective of berth allocation problems. This study focuses on the discrete dynamic berth allocation problem (discrete DBAP), which aims to minimize total service time, and proposes an iterated greedy (IG) algorithm to solve it. The proposed IG algorithm is tested on three benchmark problem sets. Experimental results show that the proposed IG algorithm can obtain optimal solutions for all test instances of the first and second problem sets and outperforms the best-known solutions for 35 out of 90 test instances of the third problem set.

## 1. Introduction

Containerization has been widely adopted in global freight transportation since the 1950s. Containerization significantly reduces shipping costs and accelerates cargo handling at ports. According to UNCTAD [1], maritime transportation is an important component of the global supply chain, with more than 8.7 billion tons of goods shipped annually. Shippers and carriers benefit from their ships spending as little time harbored in port as possible. Therefore, terminal authorities strive to provide efficient and cost-effective services that maximize terminal efficiency.

The operations of a container terminal include seaside operations, yard operations, and land-side operations [2, 3]. One important issue in seaside operations is the assignment of berthing position to a defined set of ships that must be served within a defined planning horizon. This is esteemed as a key process for container terminals. Due to operational correlation, container terminal operators and ocean carrier share the common objective in minimizing the time ships spend in port. The berth allocation problem (BAP) thus arises in dealing with terminal assignments of berths to ships, and one of its major aims is to minimize the total service time, that is, waiting time plus handling time. Various types of aims/objectives pursued by the BAP exist and can be referred to as in the excellent reviews of the literature [3] for the corresponding objective functions.

The BAP can be categorized using temporal and spatial constraints [3]. In terms of temporal constraints, BAP can be static or dynamic, while in terms of spatial constraints BAP can be for discrete, continuous, or hybrid berthing spaces. The static berth allocation problem (SBAP) disregards ship arrival time. That is, ships arrive before berth allocation is planned. The dynamic berth allocation problem (DBAP) assumes ships can arrive at any time with future arrival information being known; ships cannot be berthed before their arrival time. It should be mentioned that the DBAP solved assumes all data (including arrival times) are known in advance and therefore no reoptimization is required.

In the discrete BAP, the quay is divided into a set of berths, each of which can harbor only one ship at a time. In the continuous BAP, the quay is not partitioned into definitive berths, and a vessel can occupy any arbitrary position on the quay. This improves utilization of quay space at the cost of greater computational complexity. In the hybrid BAP, the quay is partitioned into berths, but large ships may need multiple berths, while small ships require only one berth.

The DBAP is known to be NP-hard [4] and thus is generally solved using metaheuristics. The iterated greedy (IG) algorithm is a very effective and efficient metaheuristic algorithm [5]. The major advantages of the IG algorithm are its simplicity and its extension property to be applicable to different problems. IG has exhibited state-of-the-art performance for numerous problems [6–8]. Therefore, this study proposes an IG algorithm to solve the discrete DBAP.

The remainder of the paper is organized as follows.  Section 2 describes the discrete DBAP and reviews the relevant literature.  Section 3 presents the proposed IG algorithm for tackling the discrete DBAP.  Section 4 depicts computational experiments and discusses the results. Finally, Section 5 presents concluding remarks.

## 2. Literature Review

Several mixed integer programming (MIP) models for discrete DBAP have been proposed in the literature. Imai et al. [9] were the first to present a discrete DBAP model which was an extension of an SBAP. Decision variables were used to assign ships to berths and scheduled their processing sequence in each assigned berth. Cordeau et al. [4] formulated the problem as a multidepot vehicle routing problem with time windows (MDVRPTW). Their model included different positions along the berth that resulted in different handling times for each ship.

Christensen and Holst [10] modeled the discrete DBAP as a generalized set-partitioning problem (GSPP) which assumed that the time measurements were integers (discrete time periods). In their model a column represented a feasible assignment of a single ship to a specific berth at a specific time. Buhrkal et al. [11] proposed a heterogeneous vehicle routing problem with time window (HVRPTW) model which was a simplified version of MDVRPTW. The HVRPTW model defined the problem on a graph rather than on a multigraph as shown in Cordeau et al. [4] and left the complexity of the problem unchanged. Furthermore, Buhrkal et al. [11] proposed an improved HVRPTW model, denoted as HVRPTW+, by considering break symmetry, variable fixing, and adding valid inequalities to reduce the computation time of HVRPTW. Based on various existing models, the GSPP model optimized solutions of the ship assignment problem [11]. However, the GSPP was still NP-hard. The number of columns became excessive when a large number of berthing time and ship were involved [11]. Furthermore, Vacca et al. [12] developed an exact algorithm for the integrated planning of berth allocation and quay crane assignment. Their exact algorithm could solve the BAP + QCAP (berth allocation problem and quay crane assignment problem) to optimality on the same set of instances of [4] using branch-and-price. Because of the complex nature of the problem, global optimal solutions may be difficult to obtain when the problem involves a large amount of variables. Therefore, researchers have been seeking efficient approximation algorithms that obtain near-optimal solutions reasonably fast.

Lai and Shih [13] developed a heuristic algorithm for solving the DBAP by considering the first-come-first-served rule and evaluated three different berthing policies using simulation experiments. Brown et al. [14, 15] explored berth allocation models that allow multiple ships to occupy a single berthing position. Imai et al. [16] formulated an SBAP as a nonlinear integer programming model to minimize the weighted objective of two conflicting criteria: berth performance and berthing satisfaction. Imai et al. [9] introduced a dynamic version of the problem, where each ship had a given arrival time and could only receive service after arrival. The objective was to minimize the accumulative service time of all ships.

Nishimura et al. [17] extended the DBAP to the multiwater depth configuration in a public berth system and proposed a genetic algorithm (GA) to solve the problem. Imai et al. [18] extended the DBAP to assign service priorities to ships and developed a GA-based algorithm to solve the extended problem. Cordeau et al. [4] considered a DBAP with time windows and proposed a Tabu search algorithm to solve it. Monaco and Sammarra [19] derived a more compact formulation than that of Imai et al. [9] and solved it by using a Lagrangian relaxation algorithm and a nonstandard multiplier adjustment method. Imai et al. [20] formulated a BAP that allowed a single berth to simultaneously serve two ships and solved it using GAs. Imai et al. [21] used the Lagrangian relaxation with subgradient optimization and the GA to identify noninferior solutions in a biobjective BAP model which minimized the service and delay time.

Mauri et al. [22] proposed a population training algorithm with linear programming (PTA/LP) to solve discrete DBAP. PTA/LP improved incoming columns in the column generation problem. Hansen et al. [23] presented a minimum cost berth allocation problem (MCBAP) based on an extension of the model previously proposed by Imai et al. [18] and developed a variable neighborhood search algorithm to solve the problem. Imai et al. [24] studied a variant of the DBAP in which an external terminal could be available when the port ran out of berth capacity.

Barros et al. [25] formulated a berth allocation model with tidal time windows, where berths could only be operated when the tide allowed. Their simulation model was based on the dynamic berth allocation model of Imai et al. [9]. De Oliveira et al. [26] proposed an approach based on clustering search (CS) with simulated annealing mechanism. CS was an iterative method which divided the search space into clusters and comprised a metaheuristic for solution generation, a grouping process, and a local search algorithm. Lalla-Ruiz et al. [27] proposed a Tabu search (T^2^S*) approach and a Tabu search with path relinking (T^2^S* + PR) approach to solve the discrete DBAP. T^2^S* was an improved version of T^2^S [4] that employed different neighborhood structure, and T^2^S* + PR added the path-relink techniques to the T^2^S*. T^2^S, T^2^S*, and T^2^S* + PR were tested using the instances from Cordeau et al. [4] and the newly generated problem set. The experimental results showed that the T^2^S* + PR approach was competitive with GSPP in small-scale problems and outperformed T^2^S and T^2^S*. Lin and Ting [28] proposed two versions of simulated annealing (SA) algorithm to solve the discrete DBAP. The results showed that both versions obtained the same solutions as those of GSPP and outperformed the T^2^S, PTA/LP, and CS approaches. Furthermore, the version of SA algorithm with restarting strategy (SA_RS_) outperformed that without restarting strategy (SA_WRS_).

## 3. Development of the Proposed Iterated Greedy Heuristic

A generic IG algorithm usually starts from an initial solution *π*_0_ and then generates a sequence of solutions by iterating the greedy method through the* destruction* and* construction* phases [5]. The* destruction* phase obtains a partial candidate solution *π*_*P*_ by removing a fixed number (*α*) of elements from the current candidate solution *π*. In the subsequent* construction* phase, a greedy constructive approach is used to sequentially insert the removed elements into the partial solution (*π*_*P*_) until a full solution is reconstructed. Once a complete solution is reconstructed, an acceptance criterion is applied to determine whether the new solution should replace the existing one. The process iterates through the* destruction* and* construction* phases until reaching the termination conditions. Additionally, another local search method may be applied before both the main loop and acceptance test to improve the initial solution and the reconstructed solution.

Based on the framework of the generic IG algorithm, the following subsection further discusses the solution representation, the objective function calculation, and the main steps of the proposed IG algorithm.

### 3.1. Solution Representation and Objective Function Calculation

**Figure 1:**
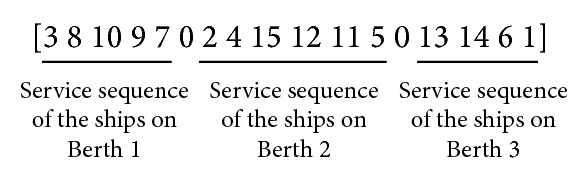
An example of the solution representation with 3-berth and 15-ship.

A solution can be represented by a numerical sequence that consists of a permutation of *n* ships and *m* − 1 zeros, where *m* denotes the number of berths. That is, the numerical sequence contains *m* segments separated by “zeros,” where each segment corresponds to the service sequence of certain ships on an assigned berth.  Figure 1 represents an example of a solution, which is explained as follows: 15 ships are to be processed on three berths, and the service sequences of the ships on berths 1, 2, and 3 are 3-8-10-9-7, 2-4-15-12-11-5, and 13-14-6-1, respectively.

The completion time of each ship on an assigned berth is calculated according to its arrival time, the sequence in the berth, and the availability of the berth. The service time of each ship is obtained by subtracting its arrival time from its completion time. Finally, the total service time can be calculated by summing up the service times of all ships.

### 3.2. Main Steps of the Proposed IG Algorithm

The main steps of the proposed IG algorithm are as follows.

**Step 1 (generate the initial solution).**

The initial solution *π* is generated using the first-come-first-served rule, which is usually implemented in real world operations. That is, the ships are sorted in ascending order of their arrival times. Each ship is then sequentially assigned to the berth with the earliest completion time of the ship that has been assigned to it. In the event of a tie, the berth with the shortest waiting time of the assigned ship is chosen. If the tie persists, the berth with the smallest number will be selected. The obtained initial solution is set as the incumbent solution π* and the best solution *π*_best_*.

**Step 2 (destruction and construction phases).**

Consider the following.

- Randomly choose *α* distinct ships from the *n* ships of π*. The value of *α* is selected randomly between *α*_min⁡_ and *α*_max⁡_, where *α*_min⁡_ and *α*_max⁡_ are the minimal and maximal numbers of unrepeated ships to be removed, respectively. Subtract these numbers from π* and add them to *π*_D_* in the order in which they are chosen, where *π*_D_* is a permutation list of the *α* removed ships.
- Sequentially reinsert the ships of *π*_D_* into *π*_P_* until a complete solution *π*_ new _* is obtained, where *π*_P_* is the partial sequence of π* obtained after removing *α* ships. When inserting a ship into *π*_P_*, all possible positions in all berths of the incumbent partial solution are considered. The best position is then chosen and recorded as the incumbent partial solution.
- IF TST(*π*_new_*) − TST(*π*_best_*) < 0, THEN set *π*_best_* : = *π*_new_* and π* : = *π*_new_*;
   ELSE_IF TST(*π*_new_*) ≤ TST(π*), THEN set π* : = *π*_new_*
   ELSE_IF *r* < *e* ^(−Δ*E*/*T*)^, THEN set π* : = *π*_new_*, where *r* ∈ [0,1] is a random number, Δ*E* = TST(*π*_new_*) − TST(π*), and *T* is the temperature.

**Step 3 (stopping criteria).**

If the computational time exceeds a specified threshold, stop the algorithm.

**Figure 2:**
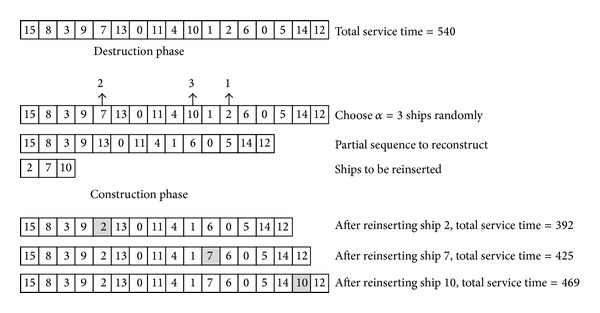
An example of IG destruction phase and construction phase.

In Step 1, an initial solution *π* is generated according to the first-come-first-served rule. Steps 2(a) and 2(b) are the* destruction* and* construction* phases, which comprise a perturbation mechanism. In Step 2(c), the Boltzmann function that is commonly used in the annealing process of SA algorithms is applied to enable the proposed IG algorithm to escape the local minimum. This is achieved by generating a random number *r* ∈ [0,1] and replacing the incumbent solution π* with *π*_new_* if *r* < *e* ^(−Δ*E*/*T*)^, where Δ*E* = TST(*π*_new_*) − TST(π*) > 0. If TST(*π*_new_*) ≤ TST(π*), the probability of replacing π* with *π*_new_* is set to one. Subsequently, the incumbent solution π* can be improved by the destruction and construction phases of Step 2 iteratively until the computational time reaching a predetermined limit. To clearly illustrate the process, Tables 1 and 2 together give a small discrete DBAP instance with 15-ship and 3-berth. The start time of berth *k*  (*s*_*k*_) and finish time of berths *k*  (*e*_*k*_) are listed in Table 1. The arrival time of ship *i* (*a*_*i*_), the end of the service time window on ship *i*  (*b*_*i*_), and the handling time of ship *i* at each berth *k*  (*t*_*i*_^1^, *t*_*i*_^2^, *t*_*i*_^3^) are given in Table 2.  Figure 2 presents an iteration of the proposed IG algorithm.

The computational complexity of the proposed IG algorithm is as follows. In Step 1, the time complexity needed for sorting the *n* ships in ascending order of their arrival times is *O*(*n*log_2_ *n*). When trying to assign each ship to the berth, there are at most *m* berths to be considered. In each iteration of Step 2, *α* distinct ships are removed from the *n* ships of current solution in the destruction phase. The computational complexity is linear. In addition, in each iteration of Step 2, *α* ships are needed to be reinserted to the partial solution in the construction phase. When trying to find the best position to reinsert the first one of *α* removed ships, there are (*n* + *m* − *α*) possible positions to be tested. Therefore, there are (*n* + *m* − *α*) times to calculate objective function value. In a similar fashion, when trying to find the best position to reinsert the *i*th ship of *α* removed ships, there are (*n* + *m* − 1 − *α* + *i*) possible positions to be tested. Therefore, the total number of calculating objective function is ∑_*i*=1_^*α*^(*n* + *m* − 1 − *α* + *i*). It should be noted that recalculation of the objective function is localized. That is, only the ships whose orders are affected by the inserted ship on the same berth are taken into account for recalculating the objective function. The proposed IG algorithm is thus adaptive and efficient.

## 4. Computational Results and Discussion

This section discusses the computational tests used to evaluate the performance of the proposed IG algorithm. The details of the test problems, parameters selection, and the computational results of the proposed IG algorithm are compared with those of the state-of-the-art algorithms, including T^2^S [4], PTA/LP [22], CS [26], T^2^S* [27], T^2^S* + PR [27], and SA_RS_ [28].

### 4.1. Test Problems

Three benchmark problem sets were used in this study. Cordeau et al. [4] provided two sets (I2 and I3) of instances that were randomly generated based on data from the port of Gioia Tauro (Italy). The I2 set includes five instance sizes: 25 ships with 5, 7, and 10 berths; 35 ships with 7 and 10 berths; and 10 instances generated for each size. The I3 set includes 30 instances with 60 ships and 13 berths. Lalla-Ruiz et al. [27] provided a new set of instances that was generated according to Cordeau et al. [4] with longer time horizon, higher traffic, and fewer available berths. New instances can be found at https://sites.google.com/site/gciports/berth-allocation-problem. This site provided nine instance sizes: 30 ships with 3 and 5 berths; 40 ships with 5, 7, and 10 berths; 55 ships with 5, 7, and 10 berths; and 60 ships with 5 and 7 berths. Ten instances are generated for each problem size.

### 4.2. Parameter Selection

The proposed IG algorithm was implemented using the C language on the Windows XP operating system and run on a personal computer with an Intel Core 2 2.66 GHz CPU and 2 G RAM. Parameter selection may influence the quality of the results. One instance was randomly selected from each size in the I2 problem set and the new problem set, and three instances were randomly selected in the I3 problem set for preliminary testing. The following combinations of parameters were tested on these instances: *T* = {0.03, 0.05, 0.10, 0.15, 0.20, 0.25} × TP/NBS; *α*_min⁡_ = 3,4, 5,6, *α*_max⁡_ = 5,6, 7,8; and *Max*⁡*T* = {0.03, 0.05, 0.10, 0.15, 0.20, 0.25} × *n* seconds, where TP denotes total processing time (∑_*i*=1_^*n*^∑_*j*=1_^*m*^ *P*_*ij*_), the summation of handling times that each ship *i* can be assigned to berth *j*, NBS is the total number of allowable ship and berth assignments, and *n* is the number of ships. For each berth, if the ship can be served, it is an allowable ship for the berth. Based on the preliminary tests, the following parameter values exhibit the best performance within a reasonable computational time: *α*_min⁡_ = 4; *α*_max⁡_ = 7; *T* = 0.05 × TP/NBS; *Max*⁡*T* is set to 0.2 × *n* seconds for sets I2 and I3, while *Max*⁡*T* is set to 0.05 × *n* seconds for the new problem set of Lalla-Ruiz et al. [27]. Therefore, these parameter values were used for all subsequent experiments in this study. For comparison with other state-of-the-art algorithms on the same base, each problem in sets I2 and I3 is solved based on 10 trials, while each problem in the new problem set of Lalla-Ruiz et al. is solved based on 30 trials.

### 4.3. Results and Discussion

Tables 3–6 list the computational results for the discrete DBAP. The optimal solution was provided by the GSPP model using CPLEX 11 [10]. CPLEX was able to get optimal solutions for all small-sized problem instances of the three benchmark problem sets. However, for part of medium-sized and all large-sized problem instances, CPLEX is terminated with memory depletion and no solution was found [27]. Tables 3, 4, and 5 list the required computation times for the GSPP model. The proposed IG algorithm is compared with SA_RS_ for the I2 problem set and results are listed in Table 3.  Table 4 lists the results of the PTA/LP, CS, SA_RS_, and IG algorithms for the I3 problem set. Furthermore, Table 5 compares the proposed IG algorithm with T^2^S*, T^2^S* + PR, and SA_RS_ for the new problem set with known optimal solutions, while Table 6 lists the results of T^2^S*, T^2^S* + PR, SA_RS_, and IG for the new problem set with unknown optimal solutions.

In Table 3, column one represents the name of the instance, while columns two and three are the optimal solutions and the computational time required by the GSPP, respectively. Furthermore, columns four to five display the best solutions obtained by SA_RS_ in 10 trials and the computational times required for SA_RS_ to obtain the optimal solutions, respectively. Columns six and seven show similar information for IG. As shown in Table 3, both the IG and SA_RS_ can obtain the optimal solutions for all instances of the I2 problem set.

Table 4 lists the computational results for the discrete case of the I3 problem set. Besides the above columns in Table 3, Table 4 lists information for PTA/LP and CS. The CS, SA_RS_, and IG obtain all optimal solutions, whereas PTA/LP cannot reach the optimal solution for all instances. In such case, PTA/LP is only 1 time unit away from optimality. Notably, if the optimal solution cannot be obtained within a certain number of trials, the computational time is de facto the maximum computational time (*Max*⁡*T* = 0.2 × *n* seconds) for these trials. This table indicates that the proposed IG algorithm is as effective as CS and SA_RS_ in optimally solving small-scale problems.

In Table 5, columns one and two represent the problem size and instance number. Columns three and four show the optimal solutions and the computational time using the GSPP. Furthermore, columns five to seven display the best solutions obtained by T^2^S* in 30 trials, the required time, and the gaps relative to the optimal solutions for T^2^S*, respectively. The gap is calculated as (Sol_*h*_ − Sol_GSPP_)/Sol_GSPP_  × 100%, where Sol_*h*_ is the solution obtained by algorithm *h* and Sol_GSPP_ is the optimal solution obtained by GPSS. Columns 8–10 listed similar information (best solutions obtained in 30 trails, the required time, and the gaps relative to the optimal solution) for T^2^S* + PR. Columns 11 to 14 show the best solutions, the average solutions, the relative gaps between best solutions to the optimal solutions, the time required to obtain the optimal solutions, and the maximal computation time for SA_RS_ in 30 trials. Similar information for the proposed IG algorithm is listed in Columns 15 to 18. The table shows that 20, 27, 30, and 30 out of 30 optimal solutions are obtained by T^2^S*, T^2^S* + PR, SA_RS_, and IG, respectively. The SA_RS_ and the proposed IG algorithm perform best for new problem sets with known optimal solutions.

Table 6 lists similar information to Table 5, except that the optimal solution is unknown and replaced by the BKS, which exhibits the best solution among the T^2^S*, T^2^S* + PR, SA_RS_, and IG approaches. For 60 problems, all solutions obtained by the proposed IG algorithm are equal to BKS, while the 13, 28, and 47 solutions obtained by T^2^S*, T^2^S* + PR, and SA_RS_ heuristic are equal to BKS.

The running time of the IG algorithm depends on various factors, including CPU, operating system, compiler, computer program, and the precision used during execution. Therefore, the relative efficiency of the algorithms is hard to determine. Tables 3–6 show the time required for GSPP, PTA/LP, CS, T^2^S*, T^2^S* + PR, SA_RS_, and IG. The GSPP formulation was implemented by generating all columns a priori using a Java program and solving the resulting integer program using CPLEX 11 (32-bit version) on a PC with an Intel Xeon 5430 (2.66 GHz) processor. T^2^S was implemented in ANSI C, and computational experiments were performed on a Sun workstation (900 MHz). Both the PTA/LP and CS were implemented using C++ and run on a PC with an AMD Athlon 64 3500 with a 2.2 GHz processor and 1 GB of RAM. T^2^S* and T^2^S* + PR were coded in Ansi C and the experiments were performed on a PC with a T4300 processor at 2.10 GHz. The computational time and results for GSPP, PTA/LP, CS, T^2^S*, and T^2^S* + PR are cited from their original papers, while the SA_RS_ is recalculated by setting the maximal computational time to be the same as that of the proposed IG algorithm. As shown in Tables 3–6, the computational time is within the acceptable range for the proposed IG algorithm.

To verify the effectiveness of the proposed IG algorithm, the proposed algorithm is compared with PTA/LP, CS, SA_RS_, T^2^S*, T^2^S* + PR, and IG by conducting paired *t*-tests on the RER (relative error rate). The RER is calculated as (TST^*h*^ − BKS*)/BKS* × 100, where TST^*h*^ denotes the total service time from algorithm *h*. The BKS* is obtained from all the compared algorithms, including those of Cordeau et al.[4], Mauri et al. [22], Buhrkal et al. [11], de Oliveira et al. [26], Lin and Ting [28], and the proposed IG approach.

At a confidence level of 95%, Tables 7 and 8 show that the proposed IG algorithm outperforms the PTA/LP in terms of the best objective value obtained for discrete DBAP (PTA/LP for the I3 set). However, IG is not statistically better than SA_RS_ and CS on the best objective value obtained, probably because these state-of-the-art algorithms are equally able to obtain the best solutions as IG. For the new data set, the proposed IG algorithm outperforms the T^2^S*, T^2^S* + PR, and SA_RS_ approaches, as shown in Table 9. This outperformance demonstrates the superiority of the proposed IG algorithm.

## 5. Conclusion

This paper studies the berth allocation problem with dynamic arrival time. Because the berth allocation problem is NP-hard, exact solution approaches cannot optimally solve realistic large-scale problems while maintaining acceptable computational complexity. An IG algorithm is proposed as an alternative method to the problem. The proposed IG algorithm is tested using three benchmark problem sets and compared with the optimal solutions (or best known solutions) from the literature. Computational results indicate that the proposed IG algorithm is effective. The proposed IG algorithm obtains all the optimal solutions of the discrete DBAP instances for the first and the second problem sets, as well as exhibiting best-known solutions for 35 out of 90 test instances in the third problem set. Future research can further examine the integration of the berth allocation and quay crane assignment problems.

## Figures and Tables

**Table 1:** Information of 3 berths.

*k*	*s*_*k*_	*e*_*k*_
1	12	300
2	12	300
3	12	300

**Table 2:** Information of 15 ships.

*i*	*a*_*i*_	*b*_*i*_	*t* ^1^_*i*_	*t* ^2^_*i*_	*t* ^3^_*i*_
1	71	300	20	20	40
2	90	300	44	44	88
3	39	300	22	22	44
4	17	300	34	34	68
5	12	300	12	12	24
6	117	300	30	30	60
7	94	300	28	28	56
8	29	300	6	6	12
9	43	300	26	26	52
10	79	300	22	22	44
11	2	300	20	20	40
12	129	300	16	16	32
13	123	300	26	26	52
14	43	300	14	14	28
15	5	300	18	18	36

**Table 3:** Computational result for I2 problem set.

Instance	GSPP		SA_RS_		IG	
	Optimal	Time	Best objective	Time to obtain the optimal	Best objective	Time to obtain the optimal
25 × 5_1	**759**	5.99	**759**	0.04	**759**	0.01
25 × 5_2	**964**	3.70	**964**	0.16	**964**	0.08
25 × 5_3	**970**	2.95	**970**	0.63	**970**	0.10
25 × 5_4	**688**	2.72	**688**	0.10	**688**	0.03
25 × 5_5	**955**	6.97	**955**	0.32	**955**	0.38
25 × 5_6	**1129**	3.10	**1129**	0.01	**1129**	0.01
25 × 5_7	**835**	2.31	**835**	0.01	**835**	0.00
25 × 5_8	**627**	1.92	**627**	0.03	**627**	0.03
25 × 5_9	**752**	4.76	**752**	0.07	**752**	0.20
25 × 5_10	**1073**	6.38	**1073**	0.59	**1073**	0.20

25 × 7_1	**657**	3.62	**657**	0.00	**657**	0.01
25 × 7_2	**662**	3.15	**662**	0.03	**662**	0.00
25 × 7_3	**807**	4.28	**807**	0.20	**807**	0.56
25 × 7_4	**648**	3.78	**648**	0.59	**648**	0.14
25 × 7_5	**725**	3.85	**725**	0.02	**725**	0.19
25 × 7_6	**794**	3.60	**794**	0.01	**794**	0.02
25 × 7_7	**734**	3.54	**734**	0.21	**734**	0.03
25 × 7_8	**768**	3.93	**768**	0.07	**768**	0.05
25 × 7_9	**749**	3.73	**749**	0.02	**749**	0.00
25 × 7_10	**825**	3.82	**825**	0.02	**825**	0.01

25 × 10_1	**713**	5.83	**713**	0.04	**713**	0.02
25 × 10_2	**727**	6.99	**727**	0.15	**727**	0.01
25 × 10_3	**761**	6.12	**761**	0.24	**761**	0.16
25 × 10_4	**810**	5.38	**810**	0.19	**810**	0.20
25 × 10_5	**840**	6.77	**840**	0.10	**840**	0.06
25 × 10_6	**689**	5.57	**689**	0.01	**689**	0.04
25 × 10_7	**666**	5.83	**666**	0.00	**666**	0.00
25 × 10_8	**855**	5.87	**855**	0.01	**855**	0.01
25 × 10_9	**711**	5.38	**711**	0.15	**711**	0.01
25 × 10_10	**801**	5.96	**801**	0.04	**801**	0.09

35 × 7_1	**1000**	12.57	**1000**	11.59	**1000**	0.37
35 × 7_2	**1192**	15.93	**1192**	9.07	**1192**	1.35
35 × 7_3	**1201**	7.16	**1201**	3.81	**1201**	0.47
35 × 7_4	**1139**	13.59	**1139**	1.65	**1139**	0.47
35 × 7_5	**1164**	11.50	**1164**	2.25	**1164**	1.26
35 × 7_6	**1686**	29.16	**1686**	8.31	**1686**	2.02
35 × 7_7	**1176**	12.89	**1176**	1.40	**1176**	0.41
35 × 7_8	**1318**	17.52	**1318**	4.95	**1318**	0.34
35 × 7_9	**1245**	8.41	**1245**	0.59	**1245**	0.25
35 × 7_10	**1109**	14.39	**1109**	7.30	**1109**	0.80

35 × 10_1	**1124**	19.98	**1124**	0.19	**1124**	0.30
35 × 10_2	**1189**	11.37	**1189**	4.47	**1189**	0.87
35 × 10_3	**938**	8.97	**938**	0.13	**938**	0.34
35 × 10_4	**1226**	10.28	**1226**	5.63	**1226**	0.50
35 × 10_5	**1349**	22.31	**1349**	0.52	**1349**	0.27
35 × 10_6	**1188**	10.92	**1188**	0.29	**1188**	0.14
35 × 10_7	**1051**	9.74	**1051**	0.21	**1051**	0.77
35 × 10_8	**1194**	9.39	**1194**	0.08	**1194**	0.06
35 × 10_9	**1311**	29.45	**1311**	0.90	**1311**	0.53
35 × 10_10	**1189**	14.28	**1189**	0.05	**1189**	0.08

Average	953.7	8.60	953.7	1.35	953.7	0.28

**Table 4:** Computational result for I3 problem set.

Instance	GSPP		PTA/LP		CS		SA_RS_		IG	
	Optimal	Time	Best objective	Time	Best objective	Time	Best objective	Time to obtain the optimal	Best objective	Time to obtain the optimal
i01	**1409**	17.92	**1409**	74.61	**1409**	12.47	**1409**	0.51	**1409**	1.53
i02	**1261**	15.77	**1261**	60.75	**1261**	12.59	**1261**	0.05	**1261**	0.11
i03	**1129**	13.54	**1129**	135.45	**1129**	12.64	**1129**	0.17	**1129**	0.28
i04	**1302**	14.48	**1302**	110.17	**1302**	12.59	**1302**	0.09	**1302**	0.32
i05	**1207**	17.21	**1207**	124.70	**1207**	12.68	**1207**	0.07	**1207**	0.07
i06	**1261**	13.85	**1261**	78.34	**1261**	12.56	**1261**	0.00	**1261**	0.00
i07	**1279**	14.60	**1279**	114.20	**1279**	12.63	**1279**	0.40	**1279**	0.54
i08	**1299**	14.21	**1299**	57.06	**1299**	12.57	**1299**	0.29	**1299**	0.71
i09	**1444**	16.51	**1444**	96.47	**1444**	12.58	**1444**	0.21	**1444**	0.57
i10	**1213**	14.16	**1213**	99.41	**1213**	12.61	**1213**	0.11	**1213**	0.18
i11	**1368**	14.13	**1369**	99.34	**1368**	12.58	**1368**	1.11	**1368**	3.29
i12	**1325**	15.60	**1325**	80.69	**1325**	12.56	**1325**	1.49	**1325**	3.90
i13	**1360**	13.87	**1360**	89.94	**1360**	12.61	**1360**	0.04	**1360**	0.07
i14	**1233**	15.60	**1233**	73.95	**1233**	12.67	**1233**	0.05	**1233**	0.09
i15	**1295**	13.52	**1295**	74.19	**1295**	13.80	**1295**	0.00	**1295**	0.10
i16	**1364**	13.68	**1365**	170.36	**1364**	14.46	**1364**	1.86	**1364**	2.89
i17	**1283**	13.37	**1283**	46.58	**1283**	13.73	**1283**	0.02	**1283**	0.07
i18	**1345**	13.51	**1345**	84.02	**1345**	12.72	**1345**	0.00	**1345**	0.10
i19	**1367**	14.59	**1367**	123.19	**1367**	13.39	**1367**	3.67	**1367**	4.24
i20	**1328**	16.64	**1328**	82.30	**1328**	12.82	**1328**	1.00	**1328**	6.37
i21	**1341**	13.37	**1341**	108.08	**1341**	12.68	**1341**	2.06	**1341**	4.27
i22	**1326**	15.24	**1326**	105.38	**1326**	12.62	**1326**	0.50	**1326**	1.18
i23	**1266**	13.65	**1266**	43.72	**1266**	12.62	**1266**	0.06	**1266**	0.12
i24	**1260**	15.58	**1260**	78.91	**1260**	12.64	**1260**	0.07	**1260**	0.39
i25	**1376**	15.80	**1376**	96.58	**1376**	12.62	**1376**	3.60	**1376**	6.45
i26	**1318**	15.38	**1318**	101.11	**1318**	12.62	**1318**	0.45	**1318**	1.33
i27	**1261**	15.52	**1261**	82.86	**1261**	12.64	**1261**	0.09	**1261**	0.28
i28	**1359**	16.22	1360	52.91	**1359**	12.71	**1359**	11.41	**1359**	11.57
i29	**1280**	15.30	**1280**	203.36	**1280**	12.62	**1280**	1.07	**1280**	5.25
i30	**1344**	16.52	**1344**	71.02	**1344**	12.58	**1344**	1.86	**1344**	2.33

Avg.	1306.8	14.98	1306.9	93.99	1306.8	12.79	1306.8	1.08	1306.8	1.95

**Table 5:** Computational result for new problem set with known optimal solutions.

Size	Instance	GSPP		T^2^S∗			T^2^S∗ + PR			SA_RS_				IG			
		Optimal	*T* (s)	Best objective	*T* (s)	Gap	Best objective	*T* (s)	Gap	Best objective	Average objective	*T* (s)	Gap	Best objective	Average objective	*T* (s)	Gap
30∗03	1	**1763**	11.25	**1763**	0.25	0.00	**1763**	0.25	0.00	**1763**	1763.00	0.04	0.00	**1763**	1763.00	0.03	0.00
	2	**2090**	22.59	**2090**	0.26	0.00	**2090**	0.26	0.00	**2090**	2090.00	0.03	0.00	**2090**	2090.00	0.05	0.00
	3	**2186**	13.68	**2186**	0.26	0.00	**2186**	0.26	0.00	**2186**	2186.00	0.04	0.00	**2186**	2186.00	0.06	0.00
	4	**1538**	12.98	**1538**	0.28	0.00	**1538**	0.28	0.00	**1538**	1538.00	0.03	0.00	**1538**	1538.00	0.02	0.00
	5	**2114**	16.12	**2114**	0.22	0.00	**2114**	0.22	0.00	**2114**	2114.00	0.05	0.00	**2114**	2114.00	0.01	0.00
	6	**2185**	36.97	2187	0.34	0.09	**2185**	0.34	0.00	**2185**	2185.00	0.05	0.00	**2185**	2185.00	0.09	0.00
	7	**1845**	23.29	1847	0.34	0.11	**1845**	0.34	0.00	**1845**	1845.00	0.31	0.00	**1845**	1845.60	0.89	0.00
	8	**1271**	9.77	**1271**	0.31	0.00	**1271**	0.31	0.00	**1271**	1271.00	0.14	0.00	**1271**	1271.00	0.13	0.00
	9	**1595**	28.25	**1595**	0.25	0.00	**1595**	0.25	0.00	**1595**	1595.00	0.06	0.00	**1595**	1595.00	0.09	0.00
	10	**2195**	9.64	**2195**	0.29	0.00	**2195**	0.29	0.00	**2195**	2195.00	0.05	0.00	**2195**	2195.00	0.05	0.00

30∗05	1	**1149**	17.93	**1149**	0.47	0.00	**1149**	0.47	0.00	**1149**	1149.53	1.02	0.00	**1149**	1149.27	0.37	0.00
	2	**1475**	30.59	1476	0.47	0.07	**1475**	0.47	0.00	**1475**	1475.53	1.08	0.00	**1475**	1475.00	0.33	0.00
	3	**1542**	26.43	**1542**	0.50	0.00	**1542**	0.50	0.00	**1542**	1542.00	0.12	0.00	**1542**	1542.00	0.07	0.00
	4	**1075**	15.72	**1075**	0.48	0.00	**1075**	0.48	0.00	**1075**	1075.00	0.14	0.00	**1075**	1075.00	0.12	0.00
	5	**1463**	23.60	**1463**	0.40	0.00	**1463**	0.40	0.00	**1463**	1463.00	0.43	0.00	**1463**	1463.00	0.03	0.00
	6	**1580**	30.84	1581	0.48	0.06	**1580**	0.48	0.00	**1580**	1580.00	0.22	0.00	**1580**	1580.00	0.19	0.00
	7	**1276**	19.39	**1276**	0.46	0.00	**1276**	0.46	0.00	**1276**	1276.00	0.25	0.00	**1276**	1276.00	0.15	0.00
	8	**870**	22.06	**870**	0.42	0.00	**870**	0.42	0.00	**870**	870.00	0.47	0.00	**870**	870.00	0.16	0.00
	9	**1134**	22.31	1153	0.50	1.68	**1134**	0.50	0.00	**1134**	1136.80	0.90	0.00	**1134**	1134.00	0.26	0.00
	10	**1527**	18.80	**1527**	0.46	0.00	**1527**	0.46	0.00	**1527**	1527.00	0.10	0.00	**1527**	1527.00	0.07	0.00

40∗05	1	**2301**	41.51	2307	0.90	0.26	2303	0.90	0.09	**2301**	2302.87	1.09	0.00	**2301**	2301.27	1.01	0.00
	2	**2829**	59.89	2835	1.09	0.21	2834	1.09	0.18	**2829**	2829.00	0.40	0.00	**2829**	2829.27	0.97	0.00
	3	**2880**	99.20	**2880**	0.50	0.00	**2880**	0.50	0.00	**2880**	2880.67	1.75	0.00	**2880**	2880.17	1.33	0.00
	4	**2001**	39.78	**2001**	0.84	0.00	**2001**	0.84	0.00	**2001**	2001.03	0.64	0.00	**2001**	2002.33	1.24	0.00
	5	**2815**	74.14	**2815**	0.76	0.00	**2815**	0.76	0.00	**2815**	2816.03	1.00	0.00	**2815**	2820.63	1.59	0.00
	6	**2934**	66.46	**2934**	0.87	0.00	**2934**	0.87	0.00	**2934**	2934.00	0.22	0.00	**2934**	2934.00	0.20	0.00
	7	**2632**	40.97	**2632**	0.79	0.00	**2632**	0.79	0.00	**2632**	2632.07	0.64	0.00	**2632**	2632.00	0.48	0.00
	8	**1835**	40.11	1836	1.28	0.05	**1835**	1.28	0.00	**1835**	1835.40	1.18	0.00	**1835**	1835.00	0.93	0.00
	9	**2086**	47.70	2095	1.07	0.43	2089	1.07	0.14	**2086**	2089.57	1.54	0.00	**2086**	2089.37	1.22	0.00
	10	**2962**	52.28	2964	1.06	0.07	**2962**	1.06	0.00	**2962**	2962.03	0.49	0.00	**2962**	2962.00	0.20	0.00

**Table 6:** Computational result for new problem set with unknown optimal solution.

Size	Instance	BKS	T^2^S∗			T^2^S∗ + PR			SA_RS_				IG			
			Best objective	*T* (s)	Gap	Best objective	*T* (s)	Gap	Best objective	Average objective	*T* (s)	Gap	Best objective	Average objective	*T* (s)	Gap
40∗10	1	**1458**	1467	1.20	0.62	1460	1.11	0.14	**1458**	1464.13	1.79	0.00	**1458**	1458.13	1.33	0.00
	2	**1375**	1381	1.01	0.44	**1375**	1.32	0.00	**1375**	1376.87	1.45	0.00	**1375**	1375.00	0.56	0.00
	3	**2119**	**2119**	0.84	0.00	**2119**	1.17	0.00	**2119**	2128.07	1.98	0.00	**2119**	2119.00	0.56	0.00
	4	**1591**	1600	1.18	0.57	1597	1.78	0.38	**1591**	1597.23	1.52	0.00	**1591**	1592.37	0.36	0.00
	5	**1847**	1849	1.11	0.11	**1847**	1.45	0.00	**1847**	1848.53	1.68	0.00	**1847**	1847.13	1.04	0.00
	6	**2080**	**2080**	0.86	0.00	**2080**	1.37	0.00	**2080**	2080.00	0.21	0.00	**2080**	2080.00	0.11	0.00
	7	**1841**	1845	1.25	0.22	**1841**	1.56	0.00	**1841**	1841.80	1.36	0.00	**1841**	1841.00	0.65	0.00
	8	**2025**	2026	1.18	0.05	2026	1.70	0.05	**2025**	2025.87	0.57	0.00	**2025**	2025.63	0.49	0.00
	9	**1880**	1888	1.06	0.43	**1880**	1.48	0.00	**1880**	1880.47	0.78	0.00	**1880**	1880.00	0.25	0.00
	10	**1883**	1905	0.71	1.17	1892	1.59	0.48	1884	1889.27	0.99	0.05	**1883**	1883.10	0.26	0.00

55∗05	1	**4689**	4693	1.65	0.09	**4689**	2.82	0.00	**4689**	4689.13	1.02	0.00	**4689**	4689.07	1.21	0.00
	2	**5467**	5483	1.37	0.29	**5467**	2.81	0.00	**5467**	5467.13	0.98	0.00	**5467**	5472.53	2.01	0.00
	3	**5499**	**5499**	1.92	0.00	**5499**	2.67	0.00	**5499**	5499.00	0.37	0.00	**5499**	5499.00	0.23	0.00
	4	**4165**	4189	1.76	0.58	4179	3.65	0.34	**4165**	4170.37	1.32	0.00	**4165**	4171.80	1.79	0.00
	5	**5478**	5484	1.39	0.11	**5478**	2.73	0.00	**5478**	5478.00	0.67	0.00	**5478**	5478.07	0.80	0.00
	6	**5595**	5599	1.45	0.07	**5595**	2.56	0.00	**5595**	5595.27	0.90	0.00	**5595**	5595.00	0.22	0.00
	7	**4870**	4902	1.90	0.66	4882	3.82	0.25	**4870**	4878.47	1.34	0.00	**4870**	4877.57	1.32	0.00
	8	**3552**	3565	1.54	0.37	**3552**	2.79	0.00	**3552**	3552.50	1.25	0.00	**3552**	3562.40	2.40	0.00
	9	**4273**	4277	1.67	0.09	4275	2.75	0.05	**4273**	4276.20	2.46	0.00	**4273**	4273.47	1.41	0.00
	10	**5739**	5739	1.85	0.00	**5739**	2.65	0.00	**5739**	5739.00	0.40	0.00	**5739**	5739.00	0.22	0.00

55∗07	1	**2846**	**2846**	3.60	0.00	**2846**	4.78	0.00	**2846**	2853.73	2.56	0.00	**2846**	2846.97	2.09	0.00
	2	**2883**	2887	3.03	0.14	**2883**	4.94	0.00	**2883**	2888.63	2.70	0.00	**2883**	2883.13	1.63	0.00
	3	**3825**	3840	4.31	0.39	3833	5.57	0.21	3831	3837.07	2.57	0.16	**3825**	3829.70	1.33	0.00
	4	**2953**	2977	2.51	0.81	2971	4.31	0.61	2953	2966.73	1.64	0.07	**2951**	2955.50	0.70	0.00
	5	**3797**	3803	3.75	0.16	3801	3.56	0.11	**3797**	3799.73	1.49	0.00	**3797**	3797.40	0.44	0.00
	6	**3783**	**3783**	2.59	0.00	**3783**	3.70	0.00	**3783**	3783.00	0.68	0.00	**3783**	3783.00	0.72	0.00
	7	**3774**	**3774**	2.43	0.00	**3774**	3.84	0.00	**3774**	3774.00	1.10	0.00	**3774**	3774.00	0.63	0.00
	8	**3862**	3864	2.09	0.05	3863	3.95	0.03	**3862**	3864.17	2.18	0.00	**3862**	3862.93	1.85	0.00
	9	**3591**	3597	3.03	0.17	**3591**	5.26	0.00	**3591**	3592.93	2.39	0.00	**3591**	3591.93	2.27	0.00
	10	**3623**	3658	2.61	0.97	3635	4.73	0.33	3630	3638.07	2.33	0.19	**3623**	3633.60	1.42	0.00

55∗10	1	**2742**	2745	8.09	0.11	2745	7.31	0.11	2744	2747.70	2.50	0.07	**2742**	2744.90	1.70	0.00
	2	**2527**	2549	8.23	0.87	2534	6.10	0.28	**2527**	2535.20	1.98	0.00	**2527**	2528.90	1.06	0.00
	3	**2544**	2545	6.20	0.04	2545	6.53	0.04	**2544**	2549.57	2.38	0.00	**2544**	2552.17	2.36	0.00
	4	**3315**	**3315**	7.00	0.00	**3315**	5.59	0.00	**3315**	3316.13	1.83	0.00	**3315**	3321.23	2.63	0.00
	5	**3109**	3147	7.66	1.22	3123	6.12	0.45	3111	3120.10	1.57	0.06	**3109**	3109.20	0.24	0.00
	6	**2283**	**2283**	6.48	0.00	**2283**	6.54	0.00	**2283**	2283.07	0.84	0.00	**2283**	2283.00	0.38	0.00
	7	**2144**	2146	5.04	0.09	2146	9.17	0.09	**2144**	2150.67	2.70	0.00	**2144**	2144.00	0.83	0.00
	8	**2720**	2743	4.98	0.85	2726	5.18	0.22	**2720**	2725.13	1.38	0.00	**2720**	2720.03	0.73	0.00
	9	**2149**	2162	6.50	0.60	2162	6.50	0.60	2152	2158.53	1.35	0.14	**2149**	2154.13	0.42	0.00
	10	**2814**	2815	5.45	0.04	2815	6.05	0.04	**2814**	2814.57	1.08	0.00	**2814**	2820.07	1.86	0.00

60∗05	1	**5753**	5761	1.99	0.14	**5753**	3.12	0.00	**5753**	5753.70	1.57	0.00	**5753**	5753.00	0.96	0.00
	2	**6884**	**6884**	2.67	0.00	**6884**	3.20	0.00	**6884**	6884.00	0.81	0.00	**6884**	6884.00	0.42	0.00
	3	**6780**	6782	2.17	0.03	**6780**	4.25	0.00	**6780**	6780.00	0.82	0.00	**6780**	6780.00	0.74	0.00
	4	**5092**	5105	2.30	0.26	5105	2.30	0.26	**5092**	5101.87	1.75	0.00	**5092**	5100.57	2.08	0.00
	5	**6715**	**6715**	2.47	0.00	**6715**	3.18	0.00	**6715**	6715.00	0.60	0.00	**6715**	6715.00	0.66	0.00
	6	**6616**	6618	2.45	0.03	**6616**	3.53	0.00	**6616**	6616.33	1.36	0.00	**6616**	6616.00	0.50	0.00
	7	**6011**	**6011**	2.66	0.00	**6011**	4.75	0.00	**6011**	6011.00	0.60	0.00	**6011**	6014.53	1.73	0.00
	8	**4385**	4406	2.64	0.48	**4385**	3.77	0.00	**4385**	4385.00	0.54	0.00	**4385**	4396.90	2.36	0.00
	9	**5235**	**5235**	2.17	0.00	**5235**	3.99	0.00	**5235**	5238.20	2.84	0.00	**5235**	5235.73	1.93	0.00
	10	**7255**	7281	2.22	0.36	7281	3.62	0.36	**7255**	7255.00	0.12	0.00	**7255**	7255.00	0.06	0.00

60∗07	1	**3707**	3724	4.40	0.46	3715	9.26	0.22	3709	3714.73	2.12	0.05	**3707**	3707.77	0.74	0.00
	2	**4147**	4191	6.39	1.06	4172	6.70	0.60	4150	4166.30	1.64	0.07	**4147**	4151.87	0.94	0.00
	3	**4273**	4290	6.78	0.40	4281	5.90	0.19	4275	4282.30	2.14	0.05	**4273**	4274.90	1.56	0.00
	4	**3910**	3916	5.32	0.15	3916	7.15	0.15	3911	3914.00	1.68	0.03	**3910**	3911.67	0.73	0.00
	5	**4251**	4264	3.99	0.31	4261	6.23	0.24	**4251**	4259.67	1.86	0.00	**4251**	4261.93	2.20	0.00
	6	**5727**	5731	6.56	0.07	5729	4.39	0.03	**5727**	5729.07	1.87	0.00	**5727**	5727.57	0.78	0.00
	7	**3719**	3749	6.61	0.81	3743	8.28	0.65	3721	3739.57	1.71	0.05	**3719**	3726.47	0.42	0.00
	8	**4582**	4600	6.96	0.39	4586	6.96	0.09	**4582**	4588.17	2.34	0.00	**4582**	4584.97	1.49	0.00
	9	**3979**	4011	5.39	0.80	4004	5.39	0.63	3985	3995.47	1.04	0.15	**3979**	3985.07	0.47	0.00
	10	**4107**	4125	5.66	0.44	4115	7.37	0.19	**4107**	4116.83	2.60	0.00	**4107**	4109.67	1.20	0.00

**Table 7:** Paired *t*-tests on the average RER for I2 problem set.

IG vs.	SA_RS_
Difference	0.0000
Degree of freedom	49
*t*-value	0.0000
One-tailed significance	0.5000

**Table 8:** Paired *t*-tests on the average RER for I3 problem set.

IG vs.	PTA/LP	CS	SA_RS_
Difference	2.5212	0.7791	0.0000
Degree of freedom	29	29	29
*t*-value	13.2444	6.5597	0.0000
One-tailed significance	<0.0001	<0.0001	0.5000

**Table 9:** Paired *t*-tests on the average RER for new problem set.

IG vs.	T^2^S∗	T^2^S∗ + PR	SA_RS_
Difference	0.2403	0.0984	0.0128
Degree of freedom	89	89	89
*t*-value	6.6505	5.3869	3.3017
One-tailed significance	<0.0001	<0.0001	0.0007
